# An Analysis of Genetic Predisposition to Hereditary Catalepsy in a Mouse Model of Neuropsychiatric Disorders Using Whole-Genome Sequencing Data

**DOI:** 10.32607/actanaturae.11875

**Published:** 2023

**Authors:** T. V. Andreeva, F. E. Gusev, N. A. Sinyakova, A. V. Kulikov, A. P. Grigorenko, I. Yu. Adrianova, D. V. Bazovkina, E. I. Rogaev

**Affiliations:** Center for Genetics and Life Science, Sirius University of Science and Technology, Sochi, 354340 Russian Federation; Vavilov Institute of General Genetics RAS, Department of Human Genomics and Genetics, Moscow, 119991 Russian Federation; Institute of Cytology and Genetics RAS, Department of Genetic Collection of Neuropathologies, Novosibirsk, 630090 Russian Federation; Institute of Cytology and Genetics RAS, Laboratory of Neurogenomics of Behavior, Novosibirsk, 630090 Russian Federation; UMass Chan Medical School, Department of Psychiatry, Shrewsbury, MA, 01545, USA

**Keywords:** catalepsy, mice, genome, brain, neurolysin

## Abstract

Catalepsy is a behavioral condition that is associated with severe psychopathologies, including schizophrenia, depression, and Parkinson’s disease. In some mouse strains, catalepsy can be induced by pinching the skin at the scruff of the neck. The main locus of hereditary catalepsy in mice has recently been linked to the 105–115 Mb fragment of mouse chromosome 13 by QTL analysis. We performed whole-genome sequencing of catalepsy-resistant and catalepsy-prone mouse strains in order to pinpoint the putative candidate genes related to hereditary catalepsy in mice. We remapped the previously described main locus for hereditary catalepsy in mice to the chromosome region 103.92–106.16 Mb. A homologous human region on chromosome 5 includes genetic and epigenetic variants associated with schizophrenia. Furthermore, we identified a missense variant in catalepsy-prone strains within the Nln gene. Nln encodes neurolysin, which degrades neurotensin, a peptide reported to induce catalepsy in mice. Our data suggest that Nln is the most probable candidate for the role of major gene of hereditary, pinch-induced catalepsy in mice and point to a shared molecular pathway between catalepsy in mice and human neuropsychiatric disorders.

## INTRODUCTION

Catalepsy is a natural condition characterized by a prolonged freezing reaction and the inability to correct an externally imposed awkward posture; it is a passive, defensive behavior found in most vertebrates. In humans, the defensive role of catalepsy is lost and it is a symptom of a number of severe mental and nervous diseases, such as schizophrenia and depression [1, 2].

In rodents, catalepsy can be caused by dopamine D2 receptor blockade with neuroleptics such as haloperidol or morphine [3, 4, 5, 6]. Drug-free catalepsy in mice (Supplementary file 1. Mouse with pinch-induced catalepsy (video file), see https://evolgenomics.org/catalepsy/)) can be induced by pinching the skin at the scruff of the neck [7], and there are significant differences between strains, in terms of their predisposition to this type of catalepsy. Mice of the most common inbred strains, such as C57BL/6J, DBA/2, and AKR/J, are resistant to pinch-induced catalepsy, but about 50% of CBA/Lac mice show hereditary catalepsy associated with depressive-like features and sensitivity to chronic antidepressant drug treatment [8, 9, 10, 11].

The main locus of hereditary catalepsy in mice has recently been mapped through QTL analysis to the distal part (61–70 cM) of chromosome 13 [12]. The genetic linkage was verified by the selective breeding experiment in [13] and transferring of the CBA-derived distal fragment of chromosome 13 between the D13Mit74 and D13Mit214 genetic markers to the genome of the catalepsy-resistant strain AKR. About 50% of mice of the congenic AKR.CBA-D13Mit76 strain showed severe catalepsy, similar to CBA mice [10]. The ASC/Icg (Antidepressant Sensitive Catalepsy) strain was created by selective breeding of CBA × (CBA × AKR) backcrosses for predisposition to catalepsy. Hereditary catalepsy in CBA mice was shown to come with depressive-like features and sensitivity to chronic antidepressant drug treatment [2, 9, 11, 14]. About 80–85% of ASC mice exhibited catalepsy [13], but the candidate genes for pinch-induced catalepsy in mice remain poorly understood.

Here, we performed whole-genome sequencing of the catalepsy-resistant (AKR/J) and catalepsy-prone mouse strains (CBA, AKR.CBA-D13Mit76, and ASC) in order to identify the putative candidate genes or chromosomal loci involved in the mechanisms of hereditary catalepsy in mice.

## EXPERIMENTAL PROCEDURES

**Animals**

Mice of the catalepsy-resistant (AKR/J) and catalepsy-prone (CBA/LacJ) strains which were maintained for more than 50 years at the Institute of Cytology and Genetics (Novosibirsk, Russia) and animals of the congenic AKR.CBA-D13Mit76 strain, with the CBA-derived fragment of chromosome 13 carrying the major gene of catalepsy transferred to the AKR genome and ASC strain created for hereditary predisposition to catalepsy, were used in this study. All animal experiments were approved by the Ethics Committee of the Institute of Cytology and Genetics RAS. All the experimental procedures were performed in compliance with the European Communities Council Directive dated November 24, 1986 (86/609/EEC). The animals were tested for catalepsy (including the AKR/J strain for the absence of catalepsy) as described earlier [15]. The test was regarded as positive if the mouse retained the imposed posture for at least 20 s. The animals with a positive pinch response (except for the control line AKR) were selected.

**Whole-genome sequencing**

Genomic DNA was extracted from mouse tail fragments (3–4 mm long). One tail fragment was used for DNA extraction for the animals of the AKR and D13Mit76C strains, and mixtures of tail fragments from two animals were used for DNA preparation for the ASC and CBA strains. DNA was purified using QIAamp DNA Mini columns (QIAGEN, Hilden, Germany). A total of 1.5 μg of genomic DNA was used for library preparation using the TruSeq DNA Sample Preparation kit v2 (Illumina, San Diego, CA, USA). Paired-end DNA libraries with a mean insert size of 350 bp were sequenced on an Illumina HiSeq2000 platform as paired-end 101 base reads. The sequence data generated for this study were submitted to the NCBI SRA (http://www.ncbi.nlm.nih.gov/sra) under accession number PRJNA900682.

**Sequencing data analysis and statistics**

The raw data were aligned to the reference mouse genome (Grcm38/mm10) using a BWA v. 0.7.17 [16] and Sarek v.2.7.1 workflow [17]. PCR duplicates were marked using the Picard tool (http://broadinstitute. github.io/picard/). Genetic variants were called using the GATK v. 4.1.7.0 [18] and annotated using the VEP software [19]. Structural variants were predicted with Manta 1.6.0 [20]. Whole-genome sequencing on an Illumina HiSeq2000 system resulted in a mean mouse genome coverage of 17–33× (Table 1).

**Table 1:** Genome sequencing statistics for the four mouse strains

Sample (strain)	Total reads	% mapped Grcm38/mm10	Mean genome coverage
HT76	561995272	99.56	19 ×
ASC	720038088	99.35	23 ×
CBA	555706906	97.95	17 ×
AKR	986011514	99.49	33 ×

## RESULTS AND DISCUSSION

**Fine mapping of the main cataleptic locus**

It was shown previously that hereditary catalepsy is a homozygous recessive condition [8]; therefore, we used only the homozygous variants that were found in the distal fragment of chromosome 13 in mice of all three catalepsy-prone strains (CBA, D13Mit76C, and ASC), but were absent in the AKR mouse strain, to search for the candidate catalepsy genes in this region.

Earlier, the main gene of catalepsy was mapped on the terminal fragment of mouse chromosome 13; the CBA-derived fragment of this chromosome, marked with D13Mit76, was then transferred to the genome of AKR, and the AKR.CBA-D13Mit76 recombinant line was created. The major gene of catalepsy was mapped further between 105.8 and 115.3 Mb of mouse chromosome 13 using the microsatellite mapping technique [10]. However, because microsatellite mapping is not sufficiently precise, the boundaries of the transferred fragment could be different. In order to remap the previously identified main catalepsy locus, we analyzed the distribution of parental (AKR and CBA-specific) homozygous variants in both the ASC and AKR.CBA-D13Mit76 strains on the distal fragment of chromosome 13 and found homozygous CBA-specific variants at the 103.92–106.16 Mb fragment in both the ASC and AKR.CBA-D13Mit76 strains.

We have determined that this locus is homologous to the human chromosome 5 region between 63.24 Mb and 65.93 Mb (according to the human reference genome GRCh38) and have screened this locus against a database of human GWAS [21]. Among others, we observed several statistically significant associations with educational attainment and cognitive performance, but also nominally significant associations with schizophrenia and major depressive disorder (Supplementary table S1. Genetic associations in the human genome region homologous to the main catalepsy region). Next, we overlapped this region with the recently reported [22] schizophrenia brain ChIP-seq data for histone 3 lysine 27 acetylation (H3K27ac), a marker of active enhancers. We found that five out of 76 H3K27ac peaks within the locus are epigenetically dysregulated in schizophrenia, specifically in neuronal cells, and that seven out of 114 H3K27ac peaks are dysregulated in bulk brain tissue (Supplementary table S2. H3K27ac regions dysregulated in schizophrenia found in the human genome region homologous to the main catalepsy region). Overall, these data indicate a shared molecular pathway between pitch-induced catalepsy in mice and human neuropsychiatric disorders.

**Coding variants in the chromosome 13 cataleptic locus**

ing short variants (SNPs and indels) in this main locus of hereditary catalepsy. We found that 6,087 of those have an allele present in all three sequenced genomes from cataleptic strains in the homozygous state but absent in the non-cataleptic strain (Supplementary table S3. Candidate short genetic variants). We also observed 239 putative structural variants in this locus, with 21 variants specific to catalepsy strains (found in all three, but not in the non-cataleptic genome). However, neither of them affects the protein-coding sequence (Supplementary table S4. Candidate large structural variants).

**Table 2:** Missense homozygous variants in the major catalepsy locus of chromosome 13 found in all cataleptic mice genomes but not in the non-cataleptic mice genome

Variant	Existing variation	Gene	Consequence	SIFT [23]
13:104069202 T/C	rs50518036	Nln	H148R	tolerated(0.35)
13:104111134 G/A	rs51459950	Sgtb	C7Y	deleterious(0.01)
13:104111173 A/G	rs50301687	Sgtb	N20S	tolerated(0.99)
13:104174454 T/C	rs51569005	Shld3	I150V	tolerated(0.41)
13:104174470 G/T	rs219600951	Shld3	S144R	tolerated(0.68)
13:104220239 T/C	rs221133823	Ppwd1	E256G	tolerated(0.23)
13:104220303 A/G	rs49763463	Ppwd1	Y235H	tolerated(0.11)
13:104230784 G/T	rs48594661	Cenpk	V43L	tolerated(1)
13:104312759 T/A	rs45772491	Adamts6	S226T	tolerated(0.29)

For further analysis, we prioritized the variations observed in protein-encoding regions and found nine of those to affect protein-coding genes in catalepsy-prone mice strains compared with non-catalepsy AKR mouse (Table 2). We analyzed all the coding variants in the GenBank genome assemblies for 14 reference mouse strains (DBA_2J_v3, BALB_cJ_v3, A_J_v3, CBA_J_v3, C3H_HeJ_v3, AKR_J_v3, NOD_ShiLtJ_v3, FVB_NJ_v3, Mm_Celera, LP_J_v1, PWK_PhJ_v3, WSB_EiJ_v3, CAST_EiJ_v3, and C57BL/6J), including the known catalepsy-resistant (C57BL/6J, DBA/1J) and catalepsy-prone (C3H/HeJ, A/He, BALB/cLac) strains [8]. The catalepsy-prone C3H/HeJ strain carries the total list of these coding variants, just like the CBA strain does, but all variants are missing in the catalepsy-prone lines A/He, BALB/cJ, except for the Nln gene mutation. In total, a single-nucleotide variant T > C (rs50518036) in the coding region of the neurolysin gene Nln was found in CBA, AKR. CBA-D13Mit76, and ASC mice, as well as in C3H/HeJ, A/He, BALB/cJ mouse strain genome assemblies, while it's absent in the GenBank genomes of catalepsy-resistant mice strains (AKR/J, C57BL/6J, and DBA/1J).

Neurolysin is a metalloendopeptidase, which plays a role in the degradation of neurotensin and bradykinin; both are pharmacological inductors of catalepsy in mice [24, 25, 26, 27]. All catalepsy-prone strains contained histidine at position 146 of nln, while the catalepsy-resistant AKR strain contained arginine. Neurolysin has never previously been implicated in catalepsy, but this enzyme degrades neurotensin, the peptide that can induce catalepsy [24]. Consequently, the Nln gene emerges as the strongest candidate to be the major gene of hereditary pinch-induced catalepsy in mice.

## CONCLUSIONS

Whole-genome sequencing of the catalepsy-prone and catalepsy-resistant mouse strains yielded valuable data for remapping the earlier identified main locus for hereditary pinch-induced catalepsy in mice on chromosome 13 to the chromosome region between 103.92–106.16 Mb and uncovered a single, potentially causing mutation in the neurolysin gene Nln in the main locus of catalepsy on chromosome 13. The major gene of catalepsy defined about 20% of catalepsy penetrance [10]. Meanwhile, the trait penetrance was 50% in recombinant D13Mit76 mice and 80% in catalepsy-prone ACS mice. The remaining penetrance is obviously the result of other genes and/or the impact of gene regulatory regions. Whole-genome data for the mouse model of hereditary behavior-trait-related depression and schizophrenia in humans should provide another resource for further identification of the genetic and epigenetic factors behind human psychiatric disorders.
